# Cognitive fatigue in multiple sclerosis is associated with alterations in the functional connectivity of monoamine circuits

**DOI:** 10.1093/braincomms/fcab023

**Published:** 2021-03-05

**Authors:** Mara Cercignani, Ottavia Dipasquale, Iulia Bogdan, Tiziana Carandini, James Scott, Waqar Rashid, Osama Sabri, Swen Hesse, Michael Rullmann, Leonardo Lopiano, Mattia Veronese, Daniel Martins, Marco Bozzali

**Affiliations:** 1 Department of Neuroscience, Clinical Imaging Sciences Centre, Brighton and Sussex Medical School, University of Sussex, Brighton BN1 9RR, UK; 2 Neuroimaging Laboratory, Santa Lucia Foundation, 00179 Rome, Italy; 3 Centre for Neuroimaging Sciences, Institute of Psychiatry, Psychology and Neuroscience, King’s College London, London SE5 8AF, UK; 4 Fondazione IRCCS Ca' Granda Ospedale Maggiore Policlinico, University of Milan, Dino Ferrari Center, 20122 Milan, Italy; 5 Department of Nuclear Medicine, University of Leipzig, 04102 Leipzig, Germany; 6 Integrated Research and Treatment Center (IFB) Adiposity Diseases, Leipzig University Medical Center, 04103 Leipzig, Germany; 7 Department of Neuroscience “Rita Levi Montalcini”, University of Torino, 10126 Torino, Italy

**Keywords:** REACT, fatigue, neurotrasmitters, noradrenaline, functional connectivity

## Abstract

Fatigue is a highly prevalent and debilitating symptom in multiple sclerosis, but currently the available treatment options have limited efficacy. The development of innovative and efficacious targeted treatments for fatigue in multiple sclerosis has been marred by the limited knowledge of the underlying mechanisms. One of the hypotheses postulates that multiple sclerosis pathology might cause reduced monoaminergic release in the central nervous system with consequences on motivation, mood and attention. Here, we applied the recently developed Receptor-Enriched Analysis of Functional Connectivity by Targets method to investigate whether patients with high and low fatigue differ in the functional connectivity (FC) of the monoamine circuits in the brain. We recruited 55 patients with multiple sclerosis, which were then classified as highly fatigued or mildly fatigued based on their scores on the cognitive sub-scale of the Modified Fatigue Impact scale. We acquired resting-state functional MRI scans and derived individual maps of connectivity associated with the distribution of the dopamine, noradrenaline and serotonin transporters as measured by positron emission tomography. We found that patients with high fatigue present decreased noradrenaline transporter (NAT)-enriched connectivity in several frontal and prefrontal areas when compared to those with lower fatigue. The NAT-enriched FC predicted negatively individual cognitive fatigue scores. Our findings support the idea that alterations in the catecholaminergic functional circuits underlie fatigue in multiple sclerosis and identify the NAT as a putative therapeutic target directed to pathophysiology.

## Teaser

Cercignani et al. report significantly reduced functional connectivity of noradrenaline transporter-related circuits in multiple sclerosis patients with high as compared to low cognitive fatigue. This result, obtained with a novel multimodal analysis approach, suggests that imbalances in catecholaminergic functional circuits might underlie fatigue in multiple sclerosis.

## Graphical Abstract

**Graphical Abstract:**
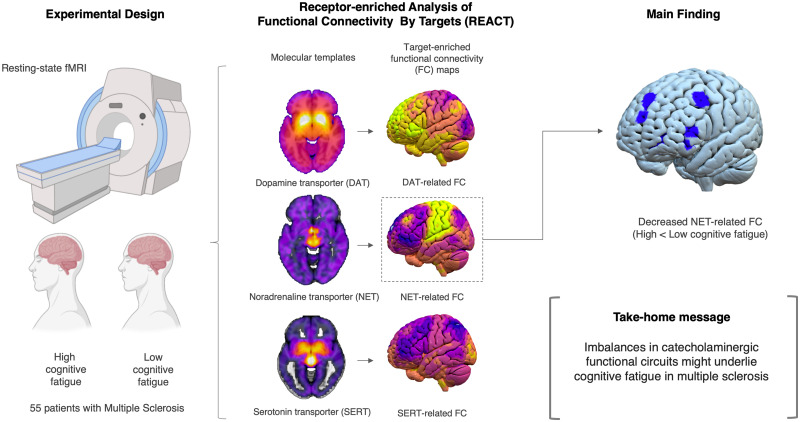

## Introduction

Fatigue is a highly prevalent and disabling symptom in multiple sclerosis,^1^ with a strong impact on patients’ quality of life.^2^ Cognitive fatigue is a subjective symptom that is typically described by patients with multiple sclerosis as a chronically present ‘mental fog’ that reduces their performance, especially—but not only—in job-related activities.^3^ The underlying mechanisms of chronic fatigue in multiple sclerosis remain largely unknown, but seem decoupled from acute neuroinflammatory episodes,^4^ which makes the management of fatigue particularly challenging.

The pathophysiology of fatigue in multiple sclerosis is still largely unknown, though different underlying mechanisms have been proposed so far.^5^ Growing evidence supports the role of aberrant monoaminergic neurotransmission.^5^^,^^6^ Monoamines are crucial modulators of functions such as motivation, mood and attention, which are all reduced in multiple sclerosis patients with fatigue. Different combinations of grey and white matter damage, which are typically observed in multiple sclerosis, might account for different patterns of chronic fatigue and inter-subject variability in response to therapies.^7^ First, both focal (i.e. brainstem monoaminergic nuclei where monoaminergic neurons are located) and diffuse grey matter pathology (i.e. cortical neurons) may reduce monoamine release or lead to poor responsiveness of neuronal targets, located mainly in the prefrontal cortex (PFC).^8–10^ Secondly, the disconnection between brainstem monoaminergic nuclei and target areas due to macro- or microscopic white matter damage may result in reduced monoaminergic release in the brainstem nuclei and/or in their projective white matter tracts.^5^ Third, inflammation may decrease monoamine synthesis or alter their function,^11^ thus lowering the neurotransmitter supply to the rest of the brain and possibly leading to a functional reorganization of central cortical networks.^12^^,^^13^

Among monoamines, a dopamine (DA) imbalance is generally considered as one of the culprits of chronic fatigue in multiple sclerosis.^6^ Supporting this idea, the two most commonly used drugs to improve fatigue in multiple sclerosis—amantadine and methylphenidate—enhance dopaminergic transmission. Although generally well tolerated, the efficacy of these drugs is limited.^7^ Hence, identifying new therapeutic targets to improve fatigue in multiple sclerosis patients remains as an unmet clinical need. This task has nevertheless been marred by the current lack of understanding of precise brain mechanisms underlying fatigue in multiple sclerosis.

While DA alterations are typically evoked to account for fatigue in multiple sclerosis, other neurochemical systems, such as noradrenaline (NA), have equally been hypothesized to contribute to fatigue more generally. The role of NA in fatigue has been investigated only in one study in Parkinson’s disease, but no significant correlations were identified between the extent of degeneration of the locus coeruleus—where NA is mainly synthetized—and the degree of fatigue.^14^ Nevertheless, the locus coeruleus projects diffusely to the entire brain (mostly PFC and cingulum) and takes a primary part in the ascending arousal system modulating arousal and attention.^10^ Moreover, the locus coeruleus regulates other higher-level cognitive processes such as working memory, motivation, pain and autonomic reflexes.^15^ Interestingly, the abovementioned drugs used to treat fatigue in multiple sclerosis are not selective for DA transmission, but also enhance NA neurotransmission. Hence, while the role of NA circuits in fatigue in multiple sclerosis has been largely overlooked, it is plausible that NA circuits may equally contribute to the genesis of fatigue and response to treatment.

Finally, preliminary studies have also suggested that a dysregulation of the serotoninergic system [serotonin (5-HT)] might contribute to the pathophysiology of fatigue in multiple sclerosis.^16^ In the more general context of fatigue (i.e. not restricted to multiple sclerosis), positron emission tomography (PET) studies have demonstrated altered 5-HT transporter distribution in patients with chronic fatigue syndrome as compared to controls, as well as in patients with Parkinson’s disease complaining of fatigue as compared to those without fatigue.^17^^,^^18^ One study using PET imaging to assess the availability of 5-HT transporters in multiple sclerosis patients when compared to controls reported a lower availability in the limbic and paralimbic regions of multiple sclerosis patients and higher availability in their frontal cortex.^19^ The same study also found a positive association between 5-HT transporters availability in the insula of multiple sclerosis patients and both their depression and fatigue scores.^19^

The neural substrates of fatigue in multiple sclerosis have been mostly studied using functional MRI (fMRI). Reduced connectivity between the basal ganglia and the PFC in multiple sclerosis patients with fatigue remains as the most consistent finding in task-related and resting-state fMRI (rs-fMRI) studies (for a review, see ref.^6^). This circuit alteration has been suggested to mostly reflect decreases in DA neurotransmission in multiple sclerosis patients with fatigue based on the known anatomy of the DA pathways. However, as fMRI has no intrinsic selectivity to any specific neurochemical target, gaining insight about the neurochemical mechanisms underlying functional alterations during disease based solely on fMRI is challenging at best. Ultimately, this technical limitation makes it impossible to guide the selection of drugs that most likely can address functional alterations as detected by fMRI.

Here, we applied the recently developed Receptor-Enriched Analysis of functional Connectivity by Targets (REACT)^20^ framework to rs-fMRI data acquired in a cohort of multiple sclerosis patients with high and low fatigue to investigate how changes in resting state functional connectivity (FC) often reported in multiple sclerosis patients with fatigue relate to the distribution of the dopamine (DAT), noradrenaline (NET) and serotonin (SERT) transporters. REACT is a multimodal approach that enriches the rs-fMRI analysis with information about the spatial distribution density of molecular targets derived from PET imaging and allows to investigate changes in FC associated with specific molecular targets. We hypothesized that some, if not all, of these transporter-enriched FC maps would show reductions in multiple sclerosis patients with higher cognitive fatigue compared to those with lower fatigue.

## Materials and methods

### Participants and study design

Seventy-one patients with relapsing-remitting multiple sclerosis were recruited from the multiple sclerosis clinic of Brighton and Sussex Universities Hospitals Trust, UK, between April 2017 and May 2018 into a larger study on multiple sclerosis fatigue. At recruitment, exclusion criteria for patients were history of other neurological diseases, or the presence of psychiatric and other clinical conditions. The depression subscale of the Hospital Anxiety and Depression Scale (HADS-D) and the Epworth Sleepiness Scale (ESS) were used to exclude participants with evidence of depression and sleep disorders at the suggested cut-off of 11 and 10, respectively.^21^^,^^22^ Participants on treatment with hypnotics within the last 4 weeks prior enrolment, on recreational drugs, or with a known alcohol abuse were excluded. Major abnormalities, such as anaemia, ongoing infections, thyroid dysfunction, vitamin deficiencies, sleep disturbances including obstructive sleep apnoea were excluded based on the blood tests performed for clinical purposes. The Brief International Cognitive Assessment for multiple sclerosis (BICAMS^23^) was used to screen for cognitive impairment. For this particular study, we also excluded patients on treatment with compounds acting on one or more of the molecular systems of interest (DA, noradrenaline, serotonin). Ethical approval was obtained from the London-Surrey Borders Research Ethics Committee (reference = 17/LO/0081). Written informed consent was obtained from all participants according to the declaration of Helsinki.

Fatigue was assessed using the Modified Fatigue Impact Scale (MFIS). The total MFIS score (MFIS-Tot; ranging 0–84) is the sum of the cognitive (MFIS-Cog), physical and psychosocial subscales. Here, we focused on MFIS-Cog. Patients were split into two groups (highly fatigued and mildly fatigued) based on their MFIS-Cog score, using the group median value as discriminator.

### Neuroimaging

MRI data were acquired on a 1.5 T Siemens Magnetom Avanto scanner (Siemens Healthineers, Erlangen, Germany) at the Clinical Imaging Sciences Centre of the University of Sussex, UK. The examination included: volumetric T1-weighted MPRAGE [echo time (TE) = 3.57 ms; repetition time (TR) = 27.30 ms; inversion time (TI) = 100ms; flip-angle = 70°; field of view = 256 × 240 mm^2^; matrix = 254 × 40; slice-thickness = 1 mm] and T2*-weighted multi-echo echo‐planar imaging^24^ for rs-fMRI (TR = 2570 ms; TE = 15, 34, 54 ms; flip-angle = 90°; resolution = 3.7 × 3.75 × 4.49 mm; matrix-size = 64 × 64; 31 axial slices; 185 volumes). T2-weighted and fluid-attenuated inversion recovery (FLAIR) scans were acquired for the purpose of identifying and quantifying white matter lesions. In addition, multi-shell diffusion-weighted MRI and quantitative magnetization transfer MRI were collected, but were not used in this study. White matter lesions were identified on FLAIR scans by two observers, and measured with local thresholding segmentation (Jim v.7, Xinapse Systems, Colchester, UK).

The rs-fMRI dataset was pre-processed using AFNI^25^ and FMRIB Software Library (FSL). Pre-processing steps included volume re-alignment, time-series de-spiking and slice time correction. After the pre-processing, functional data were optimally combined (OC) by taking a weighted summation of the three echoes using an exponential T2* weighting approach.^26^ The OC data were then de-noised with the multi-echo independent component analysis (ME-ICA) approach implemented in AFNI by the tool meica.py (Version v2.5).^27^^,^^28^ ME-ICA has proved a greater efficacy in detecting and removing motion artefacts compared to other modalities developed for single-echo data, while preserving the blood-oxygen level-dependent (BOLD) signal.^29^ White matter and cerebrospinal fluid signals were regressed out and a high-pass temporal filter with a cut-off frequency of 0.005 Hz was applied. Data were normalized into standard space, smoothed with an 8 mm^3^ Gaussian kernel and resampled at 2 × 2 × 2 mm resolution.

For the analysis with REACT, we used molecular templates of the DAT, NET and SERT systems. The DAT map is a publicly available template of ^123^I-Ioflupane single-photon emission computerized tomography (SPECT) images (https://www.nitrc.org/projects/spmtemplates) from 30 healthy subjects (HS) without evidence of nigrostriatal degeneration.^30^ The NET atlas was obtained by averaging the [^11^C]MRB PET brain parametric maps from an independent dataset of 10 HS (33.3 ± 10 years, four women).^31^ The SERT atlas is a publicly available template^32^ of [^11^C]DASB PET images of 210 healthy controls from the Cimbi database.^33^

All molecular atlases were normalized by scaling the image values between 0 and 1, although preserving the original intensity distribution of the images, and masked using a standard grey matter mask. Of note, for each atlas, we masked out the regions that were used as references for quantification of the molecular data in the kinetic models for the radioligands, namely the occipital areas for DAT and NET and the cerebellum for SERT. Finally, we resampled the SERT image in order to have all atlases in standard MNI space with 2 mm^3^ voxel size.

Details of REACT methodology can be found elsewhere.^20^ In brief, the functional circuits related to the DAT, NET and SERT systems were estimated using a two-step multivariate regression analysis^34^^,^^35^ implemented with the *fsl_glm* command of FSL. This analysis is conceptually comparable to the approach also known as dual regression, used in rs-fMRI to investigate the FC of the resting state networks. In the first step, the rs-fMRI volumes were masked using a binarized atlas derived from the molecular data to restrict the analysis to the voxels for which the transporter density information was available in the template. Then, the molecular templates were used as a set of spatial regressors to weight the rs-fMRI images and estimate the dominant BOLD fluctuation related to each molecular system at the subject level. Those subject-specific time series were then used as temporal regressors in a second multivariate regression analysis to estimate the subject-specific spatial map associated with each molecular atlas. The output consists of three maps per participant (one for each monoamine transporter system) reflecting the transporter-enriched FC. At this stage, the analysis was conducted on the whole grey matter volume. Both data and the design matrix were demeaned (–demean option); the design matrix columns were also normalised to unit standard deviation with the –des_norm option.^34^

### Statistical analysis

The subject-specific target-enriched spatial maps were compared between the two groups using permutation tests. We applied cluster-based inference within *randomise*,^36^ using 5000 permutations per test and contrast. Two contrasts were used for every kind of map, in order to test for both increases or decreases in connectivity with fatigue. A cluster was considered significant if *P*_FWE_ < 0.05, corrected for multiple comparisons using the threshold-free cluster enhancement (TFCE) option.^37^

Next, we extracted the mean FC value from the clusters showing a significant between-group difference and assessed their correlation with the individual MFIS-Cog scores. Furthermore, to gain insight about how well the transporter-enriched FC would perform in discriminating between highly fatigued and mildly fatigued multiple sclerosis patients, we also used the average of the FC values from the cluster showing the strongest association with fatigue in a receiver operating discrimination (ROC) analysis to calculate the sensitivity and specificity of this target-enriched FC-based discrimination.

### Data availability

MRI data are available from the corresponding author upon reasonable request, providing signature of an appropriate data transfer agreement. REACT is based on the tool *fsl_glm* available with FSL.

## Results

### Sociodemographic and clinical information

Two patients did not complete the MRI session and were thus excluded. Further 14 patients were excluded from the analysis because of concomitant treatment with medications that could confound DAT-, NET- and SERT-related FC connectivity (amantadine, *N* = 3; amitriptyline, *N* = 4; citalopram, *N* = 4; mirtazapine, *N* = 1; quetiapine, *N* = 1; sertraline, *N* = 4; venlafaxine, *N* = 1). The mean age of the remaining 55 patients was 42.5 (SD = 7.8) years, their median expanded disability status (EDSS) score was 1.5 (range = 0–6), and their mean HADS-D was 2.18 (SD = 2.19).

The median MFIS-Cog score was 15. Based on this value, all patients with MFIS-Cog >15 were allocated to the cognitively highly fatigued group (*N* = 26), leaving 29 in the cognitively mildly fatigued group. With the exception of two patients in the highly fatigued group and eight in the mildly fatigued group, all other patients were under disease-modifying treatment (DMTs) (Alemtuzumab: *N* = 13, Dimetylfumarate: *N* = 9, Natalizumab: *N* = 8, Teriflunomide: *N* = 4, Glatiramer Acetate: *N* = 4, Fingolimod: *N* = 4, Beta-interferons: *N* = 3). The distribution of DMTs for the two groups did not differ according to a Chi-squared test (*P*-value = 0.15). Table 1 summarizes the main demographic and clinical variables for the two groups. The mean Symbol Digit Modalities Test (SDMT) and Brief Visuospatial Memory Test Revised (BVMTR) scores were significantly lower (*P* = 0.04 and *P* = 0.05, respectively) in the fatigued when compared to the non-fatigued group. The median EDSS score, the mean HADS-D and the mean lesion volume were instead significantly higher in patients with fatigue. Hence these three variables were added as covariates to the main group comparison analysis. No between-group differences were observed for any other variables.

**Table 1:** Demographic and clinical data of the participants

	Fatigued (*N* = 26)	Non-fatigued (*N* = 29)	*P*-value
M/F	11/14	9/21	0.28^a^
Mean Age (SD)	41.9 (8.1)	43.1 (7.6)	0.6
Median EDSS (range)	2.5 (0–6)	1.25 (0–6)	**0.005** ^b^
Mean SDMT (SD)	45.00 (11.5)	51.17 (9.74)	**0.04**
Mean BVMTR (SD)	23.84 (7.34)	27.21 (5.14)	0.05
Mean CVLT (SD)	54.52 (10.18)	55.89 (11.77)	0.65
Median ESS (range)	5 (0–9)	4 (0–10)	0.4^b^
Mean HADS-D (SD)	2.84 (2.36)	1.65 (1.67)	**0.04**
Mean lesion volume (SD) (ml)	13.46 (11.82)	8.09 (5.16)	**0.03**
Mean MFIS-Cog (SD)	22.4 (5.2)	10.5 (3.8)	**<0.0001**

Statistical comparisons were performed using an independent sample *T*-test, unless otherwise specified. Boldafce values indicate statistically significant between-group differences.

a Chi-square test.

b Wilcoxon Rank Sum test.

BVMTR = Brief Visuospatial Memory Test Revised; CVLT = California verbal learning test II; EDSS = expanded disability status score; ESS = Epworth Sleepiness Scale; F = female; HADS-D = Depression subscale of the Hospital anxiety and depression scale; M = male; MFIS-Cog = Cognitive subscale of the Modified Fatigue Impact Scale; SD = standard deviation; SDMT = symbol digit modalities test.

### Multiple sclerosis patients with high fatigue present decreased frontal NET-enriched functional connectivity

**Figure 1:**
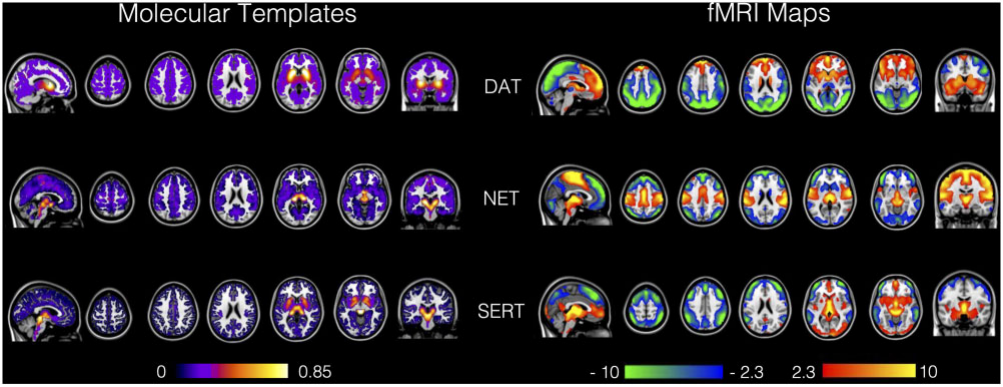
Receptor-Enriched Analysis of Functional Connectivity by Targets (REACT). PET maps used to inform REACT (left) and the resulting target-enriched functional connectivity maps, averaged across the whole study sample (right). The maps are overlaid onto the T1-weighted template in MNI space available with FSL. Note that the molecular templates have been rescaled between 0 and 1.

Figure 1 shows the molecular maps used in the dual regression and the corresponding population-averaged molecular-enriched FC maps. Note that the molecular templates have been rescaled between 0 and 1.

**Figure 2:**
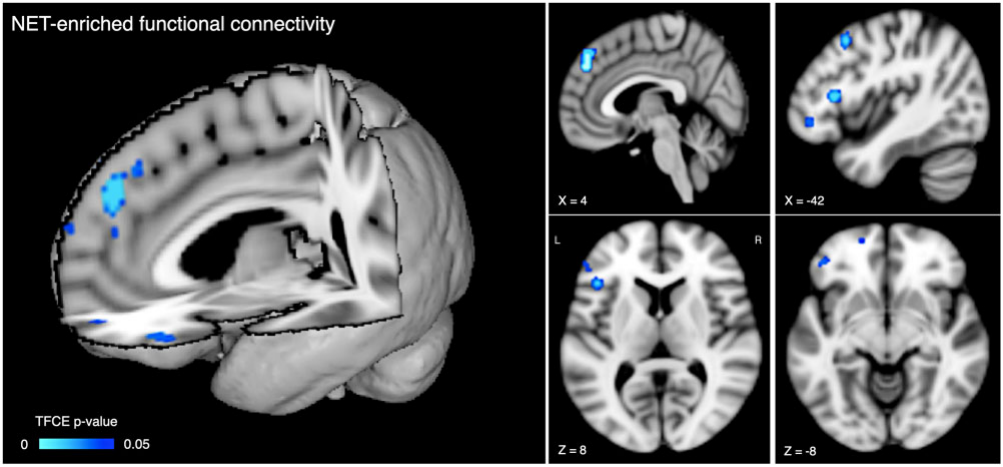
Areas of reduced noradrenaline transporter (NET)-enriched functional connectivity in multiple sclerosis patients with cognitive fatigue compared to those without. The colour scale represents the *P*-value (after correction for multiple comparisons). The thresholded statistical map is overlaid onto the MNI T1-weighted template available with FSL. The *x*, *y*, *z* values indicate the MNI coordinates of the displayed slices.

We did not find any differences between groups in the DAT-enriched and SERT-enriched maps. By contrast, we found four clusters around the mid-section in the paracingulate gyrus, and in the left hemisphere in the frontal pole, inferior frontal gyrus pars triangularis, and middle frontal gyrus where NET-enriched FC was significantly reduced (*P* < 0.05, TFCE-corrected) in highly fatigued patients compared to mildly fatigued (Fig. 2).

### NET-enriched functional connectivity predicts inter-individual variation in cognitive fatigue scores

**Figure 3:**
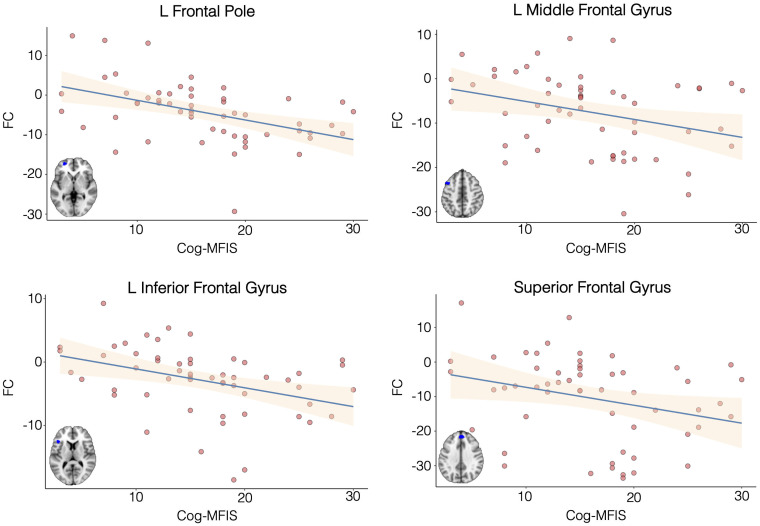
Association between noradrenaline transporter (NET)-enriched functional connectivity and inter-individual variation in cognitive fatigue scores. Scatterplots depicting negative correlations between cognitive fatigue scores and the noradrenaline transporter-enriched functional connectivity for the four clusters identified in the whole-brain analysis. Cog-MFIS = cognitive subscale of the modified fatigue impact scale; L = left.

NET-enriched connectivity values from the four clusters shown in Fig. 2 predicted negatively the MFIS-Cog scores (Fig. 3). The univariate correlation was significant for the four clusters (correlation coefficients ranging from −0.16 to −0.5; *P* values ranging from 0.03 to 5 × 10^−4^). However, a stepwise linear regression analysis suggested that the best model to explain MFIS-Cog was provided by a single regressor including NET-related connectivity in the frontal pole (coefficient = −0.42, *P* = 0.0005), with *F* = 13.79, *R*^2^ = 0.21.

### Frontal NET-enriched functional connectivity discriminates between multiple sclerosis patients with high and low cognitive fatigue with good sensitivity/specificity

**Figure 4:**
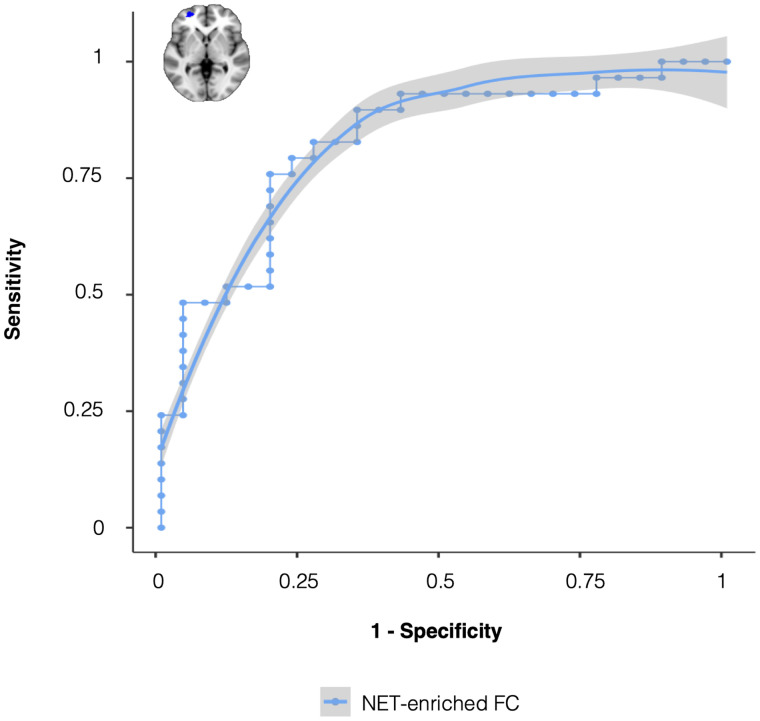
Frontal noradrenaline transporter (NET)-enriched functional connectivity discriminates between multiple sclerosis patients with and without fatigue. Receiver operating characteristic (ROC) curve for the classification of multiple sclerosis patients with and without fatigue based on the average NET-enriched functional connectivity (FC) from the significant cluster in the frontal pole.

In order to explore the ability of NET-enriched FC to discriminate between patients with high and low cognitive fatigue, we computed the ROC curve, varying the discriminating value of the FC of the frontal pole cluster between −28 and 12. The resulting curve (Fig. 4) suggests that a specificity of 0.83 could be achieved with a sensitivity just around 0.76, obtained using a FC threshold of −4.2.

## Discussion

In response to the current lack of clarity about the brain mechanisms underlying fatigue in multiple sclerosis, here we used a novel multimodal approach to investigate changes in the FC measured at rest associated with the DAT, NET and SERT circuits in multiple sclerosis patients with high fatigue as compared to those with lower levels of fatigue. Our main finding was a reduced pattern of NET-enriched FC within prefrontal cortical areas and the anterior paracingulate cortex in multiple sclerosis patients with high fatigue. Notably, the NET-enriched FC from clusters showing significant group differences could negatively predict individual MFIS-cog scores. Moreover, NET-enriched FC could discriminate between highly fatigued and mildly fatigued patients with good sensitivity and specificity.

Although no single cause for fatigue in multiple sclerosis has been identified, growing evidence supports a contribution of DA imbalance in the mesocortical pathway.^5^^,^^6^ This hypothesis stems from two empirical observations: (i) fMRI studies reported decreased connectivity between the basal ganglia and the PFC, two key-hubs of the dopaminergic mesocortical pathway^38^^,^^39^; (ii) drugs currently used in the treatment of fatigue in multiple sclerosis, such as amantadine and methylphenidate, enhance the DA neurotransmission and have been shown to reduce fatigue—although with limited efficacy. However, given the lack of intrinsic affinity of the BOLD signal for specific neurotransmitters, all previous fMRI studies could not shed light on the neurochemical systems specifically involved in the functional alterations detected in the brain of multiple sclerosis patients with high fatigue.

Our study shows for the first time that multiple sclerosis patients with high fatigue as compared to those with low fatigue show decreased connectivity in NET-related functional circuits, which we suggest might play a pivotal role in the genesis of fatigue in these patients. Importantly, these group differences on FC emerged beyond group differences on depressive symptoms, lesion load or disability, which are important confounds in studies of fatigue in multiple sclerosis. Furthermore, they cannot be explained by significant differences in DMTs distribution and average anatomical distribution of white matter lesions or any obvious brainstem lesion suggestive of a focal involvement of either the ventral tegmental area or the locus coeruleus between the two groups of patients. By contrast, we found no evidence of SERT-related FC abnormalities.^16^

At a first glance, our findings appear in direct contrast with the DA imbalance hypothesis of fatigue in multiple sclerosis. Indeed, we did not find any group differences in DAT-related FC. However, we should acknowledge that the complex biology of the NET does not allow us to exclude a contribution of DA for our findings. Indeed, the NET participates in the reuptake of both DA and NA and does so with higher affinity for DA than NA in the regions of the brain where DAT expression is low (such as in the frontal areas we found in this study).^40^^,^^41^ Hence, it is highly plausible that the decreases in NET-related FC in the frontal regions of the brain of multiple sclerosis patients with high fatigue reported here may reflect alterations in both DA and NA neurotransmission. This pattern of changes fits well with the hypothesis of disconnection in the projection pathways of both noradrenergic and dopaminergic systems in multiple sclerosis. Furthermore, these alterations match the known pharmacology of the drugs used to treat fatigue in multiple sclerosis, i.e. amantadine, methylphenidate and modafinil, which enhance both DA and NA neurotransmission. Finally, we note that frontal noradrenergic transmission has also been suggested to participate in the regulation of cognitive processes highly relevant in the context of fatigue, such as motivation.^42^^,^^43^

Our findings come with some important implications for the treatment of fatigue in multiple sclerosis. First, we provide mechanistic insights that support the rationale of using catecholamine-directed drugs to improve fatigue in multiple sclerosis as informed by physiopathology. For now, it is unclear whether the therapeutic effects of these drugs should be attributed to DA, NA or both. Based on our findings, we hypothesize that drugs such as amantadine or methylphenidate might improve fatigue in multiple sclerosis by inhibiting NET reuptake of both NA and DA in frontal circuits. Supporting this idea, in one *in* *vitro* study amantadine was shown to be about 30 times more potent in inhibiting NET than DAT.^44^ Although our study cannot clarify the mechanisms underlying treatment effects for these drugs, we showcase a useful framework to investigate such effects in future randomized, placebo-controlled, pharmaco-imaging studies.

Second, the decreased NET-related FC we report here suggests that specific inhibitors of NET reuptake, such as atomoxetine, might be of value in treating fatigue in multiple sclerosis. As far as we know, NET inhibitors have never been thoroughly investigated in the context of fatigue in multiple sclerosis. Only one open-label study in depression found that adjunctive atomoxetine improved residual fatigue.^45^ Drugs such as atomoxetine have distinct advantages over stimulants such as methylphenidate. Since atomoxetine does not affect dopaminergic neurotransmission in the basal ganglia, it is presumed to cause less anxiety, fewer motor disturbances and less potential for dependence.^46^ This hypothesis should be investigated in future clinical trials examining the clinical efficacy of NET inhibitors for fatigue symptoms in multiple sclerosis.

Third, given that we did not find any group differences on SERT-related FC, our findings suggest that drugs specifically targeting the SERT (i.e. selective serotonin reuptake inhibitors SSRIs) are unlikely to offer any promise in addressing primary fatigue in multiple sclerosis. Of course, this should not devalue the use of these drugs for addressing other psychiatric comorbidities, such as anxiety or depression. However, our findings concur with the idea that if an antidepressant is required for multiple sclerosis patients with fatigue, then dual reuptake inhibitors increasing both 5-HT and NA (i.e. venlafaxine) or NA and DA (i.e. buproprion) levels might offer some advantages over SSRIs to concomitantly improve primary fatigue.

This study also comes with some limitations. First of all, although REACT improves the specificity of FC analysis, the approach remains relatively indirect and relies on molecular templates estimated in independent cohorts of healthy individuals. Therefore, further specification from intra-regional variation across patients is not possible using the current dataset as it would require PET data for each ligand and patient. The availability of PET data from the same cohort of patients would allow the creation of patient-specific templates, which might enhance the accuracy of the maps of FC related to each target. This should be examined in future studies validating our work further. Secondly, cognitive fatigue is an ill-defined concept that can only be measured using self-reported scores. We explored the diagnostic ability of NET-enriched FC by computing the ROC curve and found that NET-enriched FC offers good sensitivity and specificity in discriminating between highly fatigued and mildly fatigued patients in our cohort. Hence, NET-enriched FC could offer a putative quantitative biomarker to identify multiple sclerosis patients with high fatigue and monitor treatment response. However, the validity of this analysis is limited by the use of the same sample for validation and testing and should be revisited in future studies using independent cohorts. Third, fatigue is often comorbid with other neuropsychiatric symptoms, such as apathy, depression or sleep disturbances. These other symptoms are important confounds in studies of fatigue in multiple sclerosis. To mitigate any potential bias, the inclusion/exclusion criteria in the present research were reasonably strict to minimise the impact of depression and sleep disturbance. Despite this, the highly fatigued group had a significantly lower average HADS-D score than the mildly fatigued group. We minimized this potential bias by adjusting all our analyses for HADS-D. Similarly, patients with high fatigue were, on average, more disabled and had larger lesion volume than the mildly fatigued group; hence, we also included these variables as covariates of no-interest. Finally, cognitive impairment was carefully checked by using the BICAMS battery. Some significant differences at the group level (*P* = 0.04) were present in the SDMT, but only six patients scored below the cut-off of 38.

In conclusion, our study supports the involvement of decreased frontal catecholaminergic connectivity, particularly that involving the NET, in the pathogenesis of cognitive fatigue in multiple sclerosis. Our findings provide further rationale for using catecholamine-enhancing drugs to treat fatigue in multiple sclerosis and uncovered a symptom-related brain mechanism through which current drugs might exert their therapeutic effects. Furthermore, we also identify NET as a putative therapeutic target directed to physiopathology, an observation that sets grounds for future trials to investigate the efficacy of specific NET reuptake inhibitors, such as atomoxetine, for fatigue in multiple sclerosis.

## Funding

M.V. and O.D. are supported by the National Institute for Health Research (NIHR) Biomedical Research Centre at South London and Maudsley NHS Foundation Trust and King’s College London.

### Competing Interests

I.B. received travel and study support from Biogen, Merck, Novartis and Sanofi-Genzyme. M.B. received travel support from Biogen and Merk, and research support from the Italian Ministry of Health. M.C. received royalties from Taylor and Francis from the publication of a book, research funding from Wellcome Trust, Motor Neuron Disease Association and the Academy of Medical Sciences. She also received institutional support from the University of Sussex and the University of Brighton. M.V. received research support from GSK. L.L. received grant support from Abbvie and Zambon and personal compensation from Abbvie, Zambon, DOC, Bial, UCB and Medtronic. T.C., W.R., O.S., S.H. and M.R. report no disclosures.

## Abbreviations

5-HT =: serotonin
BICAMS =: Brief International Cognitive Assessment for multiple sclerosis
BOLD =: blood oxygenation level dependent
BVMTR =: Brief Visuospatial Memory Test Revised
DA =: dopamine
DAT =: dopamine transporter
DMTs =: disease-modifying treatments
EDSS =: expanded disability status score
ESS =: Epworth Sleepiness Scale
FC =: functional connectivity
FLAIR =: fluid-attenuated inversion recovery
fMRI =: functional MRI
HADS-D =: Hospital Anxiety and Depression Scale
HS =: healthy subjects
ICA =: independent component analysis
MFIS =: modified fatigue impact scale
MFIS-Cog =: Cognitive subscale of the Modified Fatigue Impact Scale
NA =: noradrenaline
NAT =: noradrenaline transporter
NAT =: noradrenaline transporter
OC =: optimally combined
PET =: positron emission tomography
PFC =: prefrontal cortex
REACT =: Receptor-Enriched Analysis of Functional Connectivity by Targets
ROC =: receiver operating discrimination
rs-fMRI =: resting-state fMRI
SDMT =: Symbol Digit Modalities Test
SERT =: serotine transporter
SPECT =: single-photon emission computerized tomography
TE =: echo time
TFCE =: threshold-free cluster enhancement
TI =: inversion time
TR =: repetition time.
